# Efficacy of Oral Sirolimus Therapy for Adult Orbital Slow-Flow Vascular Malformations: A Volumetric Evaluation

**DOI:** 10.7759/cureus.71478

**Published:** 2024-10-14

**Authors:** Tadashi Nomura, Hiroshi Satake, Yuki Hata, Shunsuke Sakakibara, Hiroto Terashi

**Affiliations:** 1 Department of Plastic Surgery, Kobe University Graduate School of Medicine, Kobe, JPN; 2 Department of Plastic Surgery, Japan Community Healthcare Organization (JCHO) Osaka Hospital, Osaka, JPN

**Keywords:** adult, lymphatic malformation, mri volumetry, orbit, proptosis, sirolimus, slow-flow vascular malformation

## Abstract

Orbital lymphatic malformations significantly impair visual function, with inflammation contributing to corneal dryness and diplopia due to exophthalmos. Disease progression may lead to blindness, and in severe cases, exenteration may be required. We treated two symptomatic cases of adult orbital slow-flow vascular malformations with oral sirolimus. Although the patients maintained good visual acuity before treatment, prominent exophthalmos posed a substantial risk to visual function. After medication, eyeball proptosis improved significantly in all cases, with magnetic resonance imaging volumetry analysis showing a reduction of 28.5% to 82.1%. This treatment demonstrated minimal side effects and may serve as an initial therapeutic option.

## Introduction

Orbital lymphatic malformation (LM) is a severe clinical condition that impairs visual function. Inflammation and symptoms such as corneal dryness and diplopia are often associated with exophthalmos. If left untreated, disease progression may result in blindness, and exenteration may be necessary in some cases [1]. Microcystic LM is resistant to sclerotherapy, and surgical resection is highly challenging due to the risks of prolonged bleeding, lymph leakage, and delayed wound healing after surgery, all of which may further compromise visual function. Recent studies have demonstrated the efficacy of oral sirolimus, a mechanistic target of rapamycin (mTOR) inhibitor, in treating refractory LMs in pediatric patients [2,3]. Here, we report two cases of adult orbital slow-flow vascular malformations, including lymphatic and venous malformations, treated successfully with oral sirolimus and assessed using magnetic resonance imaging (MRI) volumetric analysis.

## Case presentation

The publication of the medical images contained in this report was approved by the patients in accordance with the ethical principles of the Declaration of Helsinki (2013 amendment).

Case 1

**Figure 1:**
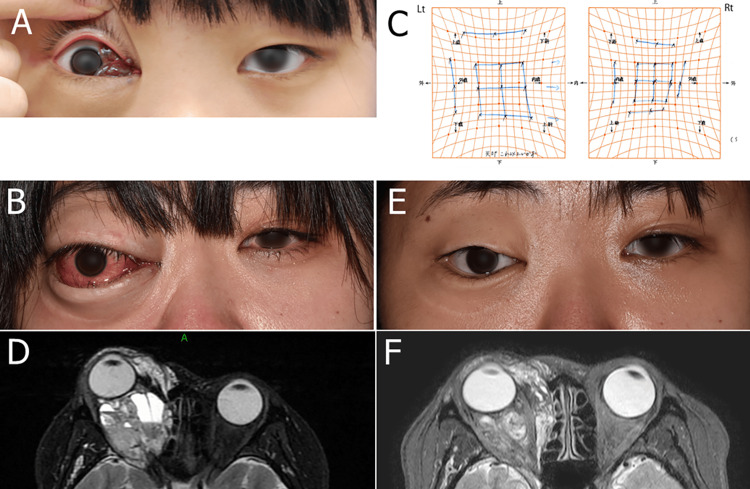
Case 1 (A) A photograph of the right eye at the age of 13 shows a blue-purple cystic lesion in the medial canthal region. (B) The patient before treatment demonstrates distinct proptosis and chemosis. (C) The Hess chart before treatment indicates a marked right eye adduction disorder. (D) A T2-weighted MRI image taken before treatment reveals a high-signal lesion with niveau formation in the posterior eyelid and muscle cone. Volumetry showed that the lesion volume was 43.9 cm³. (E) The patient, six months after starting sirolimus, shows remarkable improvement in proptosis and chemosis. (F) A T2-weighted MRI taken six months after starting sirolimus shows that the lesion volume was reduced to 31.4 cm³, indicating a reduction rate of 28.5% compared to the volume before treatment. MRI: magnetic resonance imaging

An adult female patient presented with a vascular lesion around her eye socket, first noticed in childhood and diagnosed as a venous malformation (Figure 1A). The patient, initially asymptomatic, was monitored until age 22, when the lesion began to enlarge. A partial resection of the subcutaneous lesion in the upper and lower eyelids was performed, with venous malformation as the histopathological diagnosis. However, the lesion re-expanded soon after surgery. By age 23, the patient developed marked proptosis, an inability to close her eyes during sleep, and diplopia. A decompression surgery involving the resection of an orbital floor bone through an orbital rim incision was performed by a previous plastic surgeon, and the pathological report revealed a lymphatic-venous malformation. Due to persistent symptoms, the patient was referred to our department, presenting with 15 mm of eyeball protrusion, a visual acuity of 0.7, and diplopia in the downward and inward gaze (Figures 1B-1C). MRI revealed a high-signal lesion with niveau formation in the posterior eyelid and muscle cone on T2-weighted images (Figure 1D). Blood test results showed a slight elevation in D-dimer (1.7 μg/mL), with normal C-reactive protein (CRP) (0.01 mg/dL) and white blood cell (WBC) count at 8200/μL, indicating no active inflammation or infection. Based on the clinical and laboratory findings, a diagnosis of lymphatic-venous malformation was confirmed. As the patient had previously undergone surgery, we administered oral sirolimus at a dose of 2 mg/day with the patient’s consent. The trough level of sirolimus in the blood ranged from 5.2 to 9.0 ng/mL. Following treatment, the lesion regressed quickly, allowing the patient to close her eyelid fully. Visual acuity improved to 1.2, and diplopia resolved. MRI volumetric analysis using the image analysis software OsiriX (PixMeo SARL, Geneva, Switzerland) revealed a reduction in lesion volume from 43.9 to 31.4 cm³ after six months, representing a reduction rate of 28.5%, primarily in cystic regions (Figures 1E-1F). The only side effect was mild stomatitis, which was managed successfully with topical steroids.

Case 2

A 35-year-old man presented with a three-year history of swelling in the right forehead. Following an excisional biopsy, he was diagnosed with a lymphangitic malformation. Although initially asymptomatic, three months prior to his referral to our department, the patient developed swelling of the right upper eyelid, which gradually worsened, causing pain and diplopia. Upon initial examination, the patient exhibited 6 mm of right exophthalmos, conjunctival chemosis, and a visual acuity of 0.03. MRI revealed multiple small cystic lesions on T2-weighted images below the superior rectus muscle, suspected to represent intralesional hemorrhages, with surrounding high signal intensity. Diffuse high lesions were also observed in the forehead area on T2-weighted images, consistent with microcystic LM. Blood tests showed a mildly elevated D-dimer level (1.4 μg/mL), CRP (0.02 mg/dL), and WBC count (4400/μL), with no signs of inflammation or infection (Table 1). Based on these clinical and laboratory findings, a diagnosis of mixed-type LM was made. The patient consented to oral sirolimus therapy at a dose of 2-3 mg/day. Trough sirolimus values ranged from 12.0 to 24.6 ng/mL, with dosage adjustments as needed. The lesions regressed rapidly following treatment. Six months after treatment, the diplopia and conjunctival chemosis resolved, eyelid closure was restored, and visual acuity recovered to 1.5, despite a residual 3 mm of proptosis and superficial punctate keratitis. MRI volumetric measurement using OsiriX software revealed a reduction in lesion volume from 16.8 to 3.8 cm³, representing a reduction rate of 82.1%. The only adverse effect was mild stomatitis, which resolved with topical steroid application.

**Figure 2:**
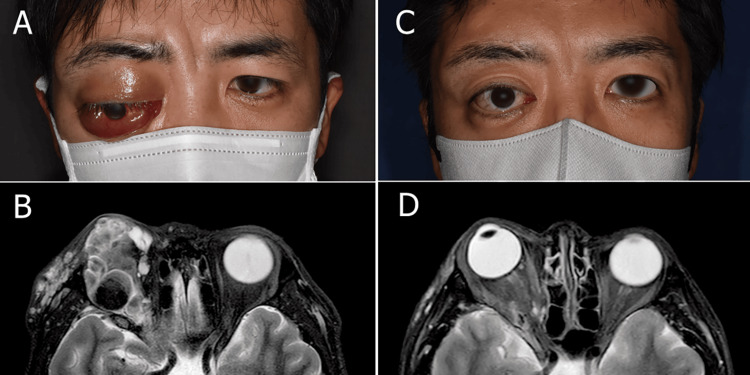
Case 2 (A) The patient before treatment showed distinct proptosis and chemosis. (B) The T2-weighted MRI image before treatment showed multiple small cystic lesions located above the superior rectus muscle, which were suspected to be intra-lesional hemorrhages, with a high signal around the lesions. Volumetry indicated that the lesion volume was 16.8 cm³. (C) The patient six months after starting sirolimus showed remarkable improvement in proptosis and chemosis. (D) The T2-weighted MRI conducted six months after starting sirolimus showed that the lesion volume was 3.8 cm³, and the reduction rate compared to before treatment was 82.1%. MRI: magnetic resonance imaging

**Table 1:** Laboratory results at the first visit WBC: white blood cell; CRP: C-reactive protein

	Case 1	Case 2	Reference value
WBC	8200	4400	3300-8600/μL
CRP	0.01	0.02	<0.14 mg/dL
Fibrinogen	296	293	200-400 mg/dL
D-dimer	1.7	1.4	<1.0 μg/mL

## Discussion

Orbital LMs may cause significant visual impairments. In a retrospective study of 42 children by Greene et al., 40% developed visual impairment, 7% became blind, and 5% required enucleation [1]. The two cases presented here were asymptomatic during childhood but developed severe symptoms in adulthood.

Microcystic LMs are generally resistant to treatment. Complete resection is challenging due to the complexity of dissection and the high likelihood of re-expansion, as well as postoperative complications such as bleeding and lymphorrhea. Local therapies, including bleomycin [4-6] and OK-432 [7,8], have shown efficacy in treating periorbital LMs. However, OK-432 can cause swelling post-treatment, especially in cases involving deep orbital or muscle cone lesions, potentially resulting in further visual impairment. Sclerotherapy may be an option for patients who are already blind before treatment, but its use is limited when the goal is to preserve vision. Furthermore, in Japan, bleomycin is not covered by public health insurance, highlighting the need for minimally invasive, effective, and sustainable treatment options.

The use of sirolimus for LMs was first reported in 2011 by Hammill et al., who demonstrated its efficacy in treating complex LMs in pediatric patients [2]. Since then, several studies have confirmed its effectiveness in managing intractable LMs, particularly in the head and neck region [9-11]. A systematic review by Teng et al. on small cystic LMs revealed long-term improvement (up to three years) in 92% of cases (46/50), with sirolimus effectively addressing symptoms such as lymphorrhea, bleeding, vesicle size, pain, and skin discoloration [12]. In a separate systematic review of orbital LMs, Shoji et al. reported a partial response in 7 out of 10 patients with vascular malformations involving lymphatic components, while three patients with microcystic lesions achieved complete resolution; 9 of these 10 patients were children [13].

To our knowledge, only two reports in the literature have described the efficacy of sirolimus in treating adult orbital LMs, both of which demonstrated improvement [14,15]. In our two adult cases, MRI volumetry showed a significant reduction in lesion volume, ranging from 28.5% to 82.1%, with clinical improvement. The anatomical structure of the orbit, enclosed by bone and narrowing with depth, may contribute to improvements in proptosis with even slight regression of the lesion. This regression may also alleviate dryness and conjunctival chemosis associated with exophthalmos. Although the precise mechanism by which sirolimus acts on orbital LMs remains unclear, it has been experimentally shown to inhibit the proliferation of LM-derived lymphatic endothelial cells [16]. This suggests that sirolimus may reduce lymphatic fluid inflow or improve drainage, although further studies are needed to confirm this hypothesis.

## Conclusions

We successfully treated two cases of adult orbital hypoplastic vascular malformations with oral sirolimus, achieving favorable outcomes. The efficacy of the treatment was objectively evaluated using MRI volumetry, which demonstrated significant lesion reduction. Given its minimal side effects, sirolimus therapy may be considered a first-line treatment, with further exploration needed in this realm.
